# Multidisciplinar treatment in postpartum depression. Coordinated attention of psychiatrist and midwife: use of desvenlafaxine while breastfeeding

**DOI:** 10.1192/j.eurpsy.2022.1439

**Published:** 2022-09-01

**Authors:** S.S. Sánchez Rus, S. Suárez-Gómez, D. Sanchez R.

**Affiliations:** 1 University Hospital Jaén, Psychiatry, Jaén, Spain; 2 Hospital José Joaquim Fernandes, Psychiatry, Beja, Portugal; 3 Hospital Universitario Insular Materno-Infantil, Puerperium Unit, Las Palmas GC, Spain

**Keywords:** postpartum depression, desvenlafaxine, multidisciplinary treatment, breastfeeding

## Abstract

**Introduction:**

Postpartum depression is a common psychiatric complication after pregnancy, so it is necessary to know the depressive symptoms to be able to carry out early prevention and treatment interventions. It is a health problem with a prevalence that ranges between 10–15% according to the world literature. Behavioral and psychosocial factors favoring postpartum depression are recognized.

**Objectives:**

-To emphasize multidisciplinary treatment in the combined attention to the mother-baby. -To demostrate decreased risk for baby if early use of antidepressants. -To evaluate of the impact of desvenlafaxine during breastfeeding.

**Methods:**

Descriptive-study. Clinical Case. Evolution of postpartum depression. Follow-up of a patient based on coordination with the midwife attending a successful breastfeeding while treatment with desvenlafaxine. Use of Edinburgh Postnatal Depression Scale.

**Results:**

-Use of Desvenlafaxine 50-100mg being compatible with breastfeeding, in adittion to depressive illness improvement *Obstetrics and psychiatry guidelines and safety considerations for lactation and antidepressants).

**Conclusions:**

-Postpartum-depression could be the first episode of depression in a healthy woman. Sometimes there are unnoticed symptoms during pregnancy. -Health-care for puerperal women should be focused on both, biomedical and psychosocial issues, with a coordinated multidisciplinar team. -Due to important early treatment during the puerperium, it is recommended a close medical control of the mother´s psychological state after the birth. -If depression appears, a psychiatric follow-up is kept even after the episode remission. Besides, the role of the midwife is essential during lactation. -Some antidepressants like desvenlafaxine have demonstrated benefits over the risk of the baby´s complications without treatment.

**Disclosure:**

No significant relationships.

